# Optimizing statistical parametric mapping analysis of ^18^F-FDG PET in children

**DOI:** 10.1186/2191-219X-3-2

**Published:** 2013-01-04

**Authors:** Frederique Archambaud, Viviane Bouilleret, Lucie Hertz-Pannier, Philippe Chaumet-Riffaud, Sebastian Rodrigo, Olivier Dulac, Francine Chassoux, Catherine Chiron

**Affiliations:** 1 Inserm, U663, Service de Neurologie et Métabolisme, Hôpital Necker, 149 rue de Sèvres, Paris, 75015, France; 2 University Paris Descartes, Paris, 75005, France; 3 CEA, I2BM, Service Hospitalier Frédéric Joliot, Orsay Cedex, 91401, France; 4 IFR 49 (Institut Federatif de Recherche), Gif sur Yvette Cedex, 91191, France; 5 Assistance Publique-Hôpitaux de Paris (AP-HP), Service de Biophysique et Médecine Nucleaire, Hôpital le Kremlin Bicêtre, Paris, 94275, France; 6 AP-HP, Service de Neuropédiatrie, Hôpital Necker, Paris, 75015, France; 7 Service de Neurochirurgie, Hôpital Sainte Anne, Paris, 75014, France

**Keywords:** PET, FDG, SPM, Children, Epilepsy

## Abstract

**Background:**

Statistical parametric mapping (SPM) procedure is an objective tool to analyze 18F-fluoro-2-deoxy-d-glucose-positron-emission tomography (FDG-PET) images and a useful complement to visual analysis. However, SPM requires a comparison to control data set that cannot be obtained in healthy children for ethical reasons. Using adults as controls showed some limitations. The purpose of the present study was to generate and validate a group of *pseudo*-*normal* children as a control group for FDG-PET studies in pediatrics.

**Methods:**

FDG-PET images of 47 children (mean ± SD age 10.2 ± 3.1 years) with refractory symptomatic (MRI-positive, *n* = 20) and cryptogenic (MRI-negative, *n* = 27) focal epilepsy planned for surgery were analyzed using visual and SPM analysis. Performances of SPM analysis were compared using two different control groups: (1) an adult control group consisting of healthy young adults (*n* = 25, 30.5 ± 5.8 years, adult PET template) and (2) a pediatric *pseudo*-*control* group consisting of patients (*n* = 24, 10.6 ± 3.1 years, children PET template) with refractory focal epilepsy but with negative MRI and with PET considered normal not only on visual analysis but also on SPM.

**Results:**

Among the 47 children, visual analysis succeeded detecting at least one hypometabolic area in 87% of the cases (interobserver kappa = 0.81). Regarding SPM analysis, the best compromise between sensitivity and specificity was obtained with a threshold of *p* less than 0.001 as an extent of more than 40 voxels. There was a significant concordance to detect hypometabolic areas between both SPM analyses [kappa (*K*) = 0.59; *p* < 0.005] and between both SPM and visual analyses (*K* = 0.45; *p* < 0.005), in symptomatic (*K* = 0.74; *p* < 0.005) as in cryptogenic patients (*K* = 0.26; *p* < 0.01). The pediatric pseudo-control group dramatically improved specificity (97% vs. 89%; *p* < 0.0001) by increasing the positive predictive value (86% vs. 65%). Sensitivity remained acceptable although it was not better (79% vs. 87%, *p* = 0.039). The main impact was to reduce by 41% the number of hypometabolic cortical artifacts detected by SPM, especially in the younger epileptic patients, which is a key point in clinical practice.

**Conclusions:**

This age-matched pseudo-control group is a way to optimize SPM analysis of FDG-PET in children with epilepsy. It might also be considered for other brain pathologies in pediatrics in the future.

## Background

Visual inspection of 18F-fluoro-2-deoxy-d-glucose-positron-emission tomography (FDG-PET) images is extensively used in pediatric clinical practice, but it remains subjective and depends on the expertise and experience of the observer. By contrast, statistical parametric mapping (SPM) is an objective and therefore observer independent method of analysis [1,2], but it requires comparison to a control data set that is quite impossible to obtain in healthy children for ethical reasons. Based on FDG-PET in pediatric epilepsy, the present study proposes to generate and validate a *pseudocontrol* group of pediatric patients for SPM studies in children.

PET with 18F-fluoro-2-deoxy-d-glucose or FDG-PET currently plays a key role in the investigation and management of patients with refractory focal epilepsy, particularly when surgery is a therapeutic option [3,4]. The use of FDG-PET over the past decades has shown that regions of interictal hypometabolism are strongly associated with the seizure onset zone (also called the epileptogenic zone) in adults as in children, although the hypometabolism may often extend beyond this zone [5-7] or occasionally miss it [8]. Findings are usually analyzed visually, and this is considered to carry powerful detecting value, although it is highly variable according to the location (temporal/extratemporal) and type (positive/negative MRI) of epilepsy [9-11]. PET/MRI coregistration also impacts visual detection [12-14]. SPM proved to be a useful strategy for FDG-PET in adults with refractory focal epilepsy not only in temporal lobe cases [5,7,15-19], but also in extratemporal epilepsy and/or negative MRI, where sensitivity of visual analysis is lower [20]. For instance, in MRI-negative frontal lobe epilepsy patients, SPM sensitivity was equivalent or superior to visual analysis [20,21]. As a result SPM is more and more used as a complementary procedure to study focal epilepsy in adults.

By contrast, SPM in children with epilepsy remains limited to a few teams [22-26] and provides discordant results [14,27]. SPM procedure requires image normalization to a template before a statistical comparison is done to a control group. Because of ethical constraints regarding PET in normal children, pediatric SPM application most often has to use an adult template and adult controls, but such a procedure has major limitations. One issue is the difference in size of the head of adults and children which cannot be solved with spatial correction and requires the use of a pediatric template for children below the age of 6 years [23]. Another issue is the major age-related changes of cerebral glucose metabolism, in cortical as in the subcortical structures, throughout the entire childhood [28,29]. Regional metabolism increases from birth to around 4 years up to values over twice the adult values, which were maintained until the end of the first decade, when they began to decline and reach adult rates by the end of the second decade. Metabolism increases earlier in the sensorimotor and occipital cortex than in the frontal cortex and increases higher in the cerebral cortex than in subcortical structures and cerebellum [28]. In the anterior cingulate cortex and the thalamus in particular, metabolism continues to increase up to 25 years and then remains relatively stable [29]. Such widespread hypometabolic changes related to the maturation of cortical and subcortical structures may complicate the interpretation of SPM results in children when compared to adults (is a given hypometabolism pathological or physiological?) and may decrease the sensitivity of detection of hypometabolic areas using SPM. These issues point out the need of an age-matched control group.

Despite continuous debate, ethical constraints prevent investigating healthy children as control subjects for PET studies. To our knowledge, only two authorizations have been delivered up to now, and they did not include patients under 6 years of age because they usually need sedation to be scanned [30,31]. As an alternative, populations of pediatric patients had therefore to be used as control groups. A first option was to focus on children with another pathology and to test the difference with the pathology to be studied. Using this approach, Zilbovicius et al. succeeded identifying bitemporal dysfunction with H_2_0^15^ PET in autistic children compared to children with idiopathic mental retardation [32], and Juhasz et al. identified a medial prefrontal hypometabolic network with FDG-PET in epileptic children with aggressive behavior compared to non-aggressive ones [33]. However, such control groups cannot be extended to other studies since they provide PET images with pathology-related anomalies. A second option was to generate pediatric control groups from patients with normal PET images. Two historical studies provided FDG-PET imaging from respectively sick children considered retrospectively as normal and pediatric idiopathic focal epilepsy, thus permitting to establish the maturational profile of cortical and subcortical metabolism, but the data are not compatible with current PET machines [28,34]. The present study provides compatible data from the epilepsy field.

The aim of this study was to evaluate a pediatric age-paired pseudo-control group compared to a classical adult control group in order to optimize the use of SPM analysis in children with refractory epilepsy.

## Materials

We retrospectively studied the 71 consecutive children referred for FDG-PET examination at our institution during a 4-year period. Inclusion criteria were the following: diagnosis of refractory focal epilepsy, previous extensive work-up including video-EEG (electroencephalogram) recordings and MR imaging, and age over 2 years (based on recruitment). The protocol was approved by the institutional ethical standards committee on human experimentation, and written informed consent was obtained from all guardians of patients participating in the study.

Clinical findings are summarized in Table 1. Patients’ age at PET ranged from 2.5 to 17.9 years. MRI had been analyzed by a trained radiologist (LHP) blind to PET findings. Finding was considered negative (cryptogenic epilepsy) in 51 cases (72%), whereas a lesion was identified (symptomatic epilepsy) in the 20 others. Most often there was a suspicion of focal cortical dysplasia (*n* = 16). Other causes were hippocampal sclerosis (*n* = 1), ischemic lesion in the white matter (*n* = 2), or extensive cerebral malformation despite focal epilepsy (*n* = 1). Lateralization and hypothesis on the location of the seizure onset zone (SOZ) was based on the scalp video-EEG in all and on the additional intracranial depth electrodes in 15 of them. Seizures were recorded in all patients but with three presenting active and permanent interictal spike focus concordant with seizure semiology. SOZ equally involved right or left hemispheres; lateralization was undetermined (bilateral in 12 cases, frontal and/or central, unknown in 1 case) in 18% of the patients. SOZ mostly affected the frontal or central regions, and the location was undetermined in only 4% of the cases. Seventeen children have currently been operated on with at least 1 year follow-up, and 13 of them are seizure-free.

**Table 1:** Clinical findings

		**All (*n *= 71)**	**Pseudo-control group (*n *= 24)**	**Patient group (*n *= 47)**
Age range (years) (mean ± SD)		2.5 to 17.9 (10.2 ± 3.6)	4.5 to 17.9 (10.6 ± 3.1)	2.5 to 17.2 (9.9 ± 3.1)
Sex	Females	38	15	23
	Males	33	9	24
MRI	Negative	51	24	27
	Positive	20	0	20
EEG	Scalp	71	24	47
	Intracranial	15	3	12
SOZ lateralization	Right	28	7	21
	Left	30	10	20
	Undetermined	13	7	6
SOZ location	Frontal	21	9	12
	Central	19	6	13
	FT	5	1	4
	Temporal	14	4	10
	TPO	7	3	4
	Occipital	2	0	2
	Undetermined	3	1	2
Epilepsy surgery	Performed	21	4	17^a^
	Follow-up >1 year	17	3	14^b^
	Seizure free at 1 year follow-up	13	1	12^b^

^a^Five patients required a second operation to complete the resection; ^b^after the last operation. SD, standard deviation; y, years; EEG, electroencephalogram; SOZ, seizure onset zone; FT, fronto-temporal; TPO, temporo-parieto-occipital.

**Figure 1:**
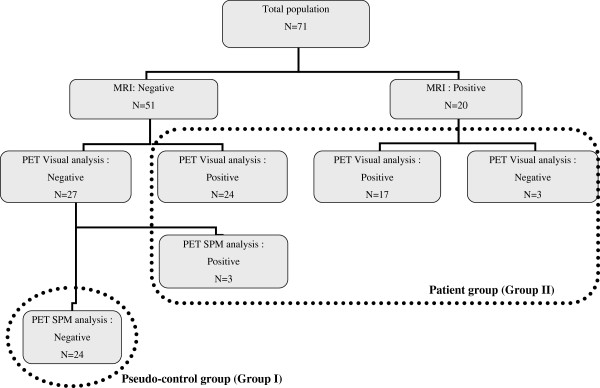
Flow chart.

We defined two groups according to MRI and PET analysis (using both visual and SPM analysis, see Methods) (Figure 1). Group I (pseudo-control group) comprised the 24 children with negative MRI and negative (normal) PET; group II (patient group) comprised the remaining 47 children with positive (abnormal) PET (20 had positive MRI, 27 had negative MRI). The clinical characteristics of groups I and II were similar regarding age, sex, and lateralization and location of the SOZ (Table 1). They were compared to an in-house adult control group (21 healthy adults, aged 21 to 40 years, mean 30.5 ± 5.8 years), in which PET scans were performed as a research protocol approved by the local ethics committee (CPP Pitié-Salpêtrière, Paris, France) and written informed consent was obtained.

## Methods

### Image acquisition

#### ^*18*^*F*-*FDG PET*

Patients were examined using ECAT EXAT HR+ (Siemens Medical Solutions, Munich, Germany) (I2BM, CEA, Hospital F. Joliot, Orsay, France), a whole-body scanner that can acquire in 3D mode 63 slices of 2.4-mm thickness simultaneously. Transverse and axial intrinsic spatial resolutions at the center of the field of view are respectively 4.3 and 4.1 mm. The patients were investigated in a fasting and resting state, in a quiet, dimly lit environment. The last seizure had occurred more than 6 h before PET examination except for 11 patients (less than 1 h in three, between 1 and 6 h in eight). In 14 patients, 5 mg/kg of pentobarbital were administered rectally for sedation 1 h before the PET examination to keep the child still during the exam, a procedure which does not impact glucose metabolism in one region specifically [35]. A brain attenuation map was obtained using a transmission acquisition of three rod sources of ^88^Ge for 15 min. Then 3.7 MBq/kg of ^18^FDG (maximum 180 MBq) was administered intravenously, and the child remained lying in the scanner. At 30 min the patients’ head was precisely re-positioned similar to transmission using face landmarks and 3D laser, and the PET dynamic acquisition began, including four frames in 5 min. Data sets were reconstructed using Hanning apodization window (0.5 cycles per pixel cut-off) as radial and axial filters providing an image resolution of 6.6 mm in the three directions.

#### MRI

Brain MRI was obtained for all subjects using a 1.5 Tesla imager (General Electric Healthcare, Buc Cedex, France) on the same day. A standard T1-weighted inversion-recovery fast spoiled gradient recalled (IR-FSPRGR) sequence was performed with axial orientation using an inversion/echo/repetition time, with a 1.2-mm slice thickness, as well as T2-weighted and fluid attenuated inversion recovery sequences in coronal and/or axial orientation.

### Image analysis

MRI images were interpreted by a pediatric neuroradiologist (LHP) who was blinded to the patients’ identity.

#### ^18^F-FDG PET visual analysis

^18^FDG-PET images were analyzed independently by two experts trained to perform ^18^F-FDG PET analysis (FA and FC) after they were given information on the supposed location of SOZ as usually done in the literature. Transaxial, sagittal, and coronal slices were analyzed using the local software BrainVisa-Anatomist (brainvisa.info/index_f.html) after reorientation in either hippocampal plane (for temporal lobe epilepsies) or CA-CP plane (for extratemporal epilepsies). The cortical areas of ^18^FDG hypofixation (hypometabolisms) were classified according to their extent (unilateral lobar, unilateral multilobar, hemispheric, and bilateral), intensity (discrete, moderate, or severe), and location among the five left and right following cortical regions systematically analyzed (ten regions per patient): frontal, central, parietal, temporal or insular, and occipital. A hypometabolic area was considered significant when seen on more than one slice (3.5-mm thickness) and had moderate to severe intensity. To assess the concordance between both experts, a kappa test was performed. In patients with discordant results, images were reviewed by both experts to reach a consensus.

#### ^*18*^*F*-*FDG PET SPM analysis*

It was achieved using MATLAB7 (The Mathworks, Inc., Natick, MA, USA) and the statistical parametric mapping software SPM5 (http://www.fil.ion.ucl.ac.uk). Individual analysis was based on the contrast patient < controls (hypometabolism), using a 2-sample *t* test and age as covariate. The resulting SPM (*t*) statistic threshold was at three levels: *p* < 0.001 (uncorrected), *p* < 0.01 (uncorrected), and *p* < 0.05 (corrected), and at three extents of contiguous voxels per cluster of 40, 100, and 200, as previously reported [27]. Similarly to the visual analysis, the significant hypometabolic clusters were classified according to their location among the ten previously mentioned regions. We defined as *true hypometabolic clusters* the significantly abnormal clusters having a cortical location concordant with visual analysis (hypometabolic areas) or being clinically relevant when visually absent (i.e., on the same side and with the same location as SOZ); the remaining abnormal clusters were defined as *artifacts*. True hypometabolic clusters and artifacts are retrospectively defined patients with *true positive* and *false positive* PET at SPM analysis (Figure 1). We also reported the hypometabolic clusters located within the interhemispheric fissure, but they were excluded from the analysis since they were extracortical.

Groups I and II were analyzed as follows (Figure 1):

Group I (*Pseudocontrol group*). To compose this group, we initially considered the 27 MRI-negative children with normal PET at visual analysis. We normalized PET images using an in-house pediatric template (44 children with negative MRI, including 28 with focal epilepsy and 16 with degenerative-like neuro-Langerhans histiocytosis, mean age 10 ± 3 years) [36]. We compared each child to the rest of the group (*n* = 26) in order to detect any residual cluster with abnormal metabolism and therefore to reach the lowest inter-subject variability as possible [37]. For this analysis we selected the threshold *p* < 0.001 and voxel size greater than 40 (see “Results”), and we also studied the contrast patient > controls (hypermetabolism) using the same parameters as for hypometabolism. True hypo and/or hypermetabolic clusters were found in three children; thus, the definite pseudo-control group was restricted to the 24 children without any relevant metabolic abnormality after this 2-step procedure.

Group II (*Patient group*). This group comprised the 44 children with abnormal PET at visual analysis and the three patients excluded from the previous group. Two successive SPM analyses (*SPM*-*A* and *SPM*-*P*) were performed using two different control groups: (1) SPM-A, PET images were normalized using the in-house adult control template and statistics was compared each of the 47 pediatric patients to this adult control group; (2) SPM-P, PET images were normalized using the same in-house pediatric template as for group I and was statistics compared each of the 47 pediatric patients to the pseudo-control group (*n* = 24).

Based on the true hypometabolic clusters and the hypometabolic artifacts on SPM, we determined the sensitivity, the specificity, and the predictive value of SPM-A and SPM-P analyses to detect significant hypometabolic areas when compared to a standard including visual analysis and clinical relevance. Sensitivity and specificity were compared between SPM-A and SPM-P using McNemar’s test.

#### Concordance between analysis procedures

It was assessed using the kappa test. The three procedures (visual, SPM-P, and SPM-A) were considered concordant when they were all negative or when the most intense hypometabolic area on visual analysis corresponded to the most significant hypometabolic cluster on SPM analyses. Concordance was complete when the number of hypometabolic regions involved was similar within the three procedures, otherwise it was partial. The procedures were considered discordant in the other conditions, i.e., when one of them did not disclose any metabolic abnormality that the two others did. We studied between-procedure concordance in the whole population of patients (group II) and in cryptogenic versus symptomatic ones.

## Results

### Visual analysis

First visual analysis found a concordance between both experts for 61 children (86%) (kappa = 0.8113, *p* < 0.05); 28 of them had no abnormality, and the remaining 33 had at least one hypometabolic area. The extent, location, and intensity of hypometabolic areas were also concordant between both experts. In nine other children, discordances involved mild to moderate hypometabolism: as a result, they were differently classified as having either no or unilateral lobar hypometabolism (five cases) and either unilateral or bilateral hypometabolism (four cases). In only one remaining child, the expert’s findings were totally discordant (right lobar against left multilobar).

**Figure 2:**
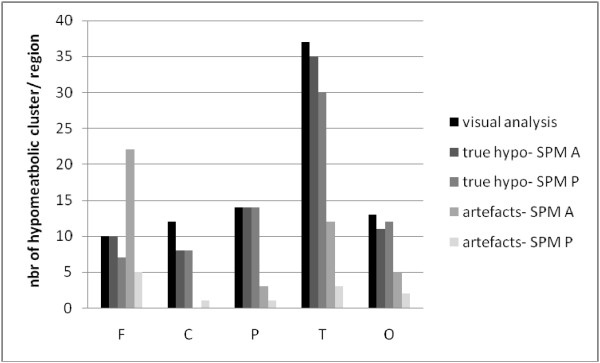
**Cortical location of the hypometabolic areas.***X* axis presents the number of hypometabolic areas using visual analysis (*n* = 86) and of hypometabolic clusters (true hypometabolic areas and artifacts) using SPM-P (*n* = 83) and SPM-A (*n* = 120), according to their regional location on the cortex: F, frontal; C, central or opercular; P, parietal; T, temporal or insular; O, occipital. *Y* axis presents the number of hypometabolic areas among the 470 regions visually analyzed (47 patients, ten regions/patient). Notice that visual analysis is equal or superior to SPM to identify a hypometabolic area in any region, and both SPM procedures are quite equivalent to identify a true hypometabolic cluster in any region, but SPM-A identifies more artifacts than SPM-P, particularly in the frontal and temporal cortices, the most frequent hypometabolic locations.

After a second visual analysis of the discordant cases, a consensus was reached: finally there were 30 children without any hypometabolism (*negative*/*normal* PET) [27 with negative MRI, the three others having either a white matter ischemic lesion (two cases) or an unusual pattern of the precentral gyrus (one case)] and 41 children (58%) with at least one hypometabolic area (*positive*/*abnormal* PET) (Figure 1). This rate reached 87% in the patient group. Extent of hypometabolism was unilateral lobar in 18 cases, unilateral multilobar in 14, hemispheric or bilateral in 9. Among the 470 regions analyzed (10 regions in 47 patients), 86 (18%) were hypometabolic, and the intensity was more often severe (64/86) than moderate (22/86). The most frequent location was temporal due to the high frequency of temporal involvement in the multilobar hypometabolic areas (Figure 2).

Among the 86 detected hypometabolic regions, 48 (56%) were concordant with the SOZ (25 in symptomatics, 23 in cryptogenics): 32 (37%) of them were superimposed with SOZ (half were temporal) and 16 others (19%) were more extended than SOZ and involved adjacent cortical areas. In 19/86 regions (22%), the location of visual hypometabolism was different from the supposed location of the SOZ (four in symptomatics, 15 in cryptogenics). In the remaining 19 regions (22%), either the SOZ location was undetermined (nine regions in 2 patients) or was bilateral with a contralateral hypometabolism (ten regions in 6 patients).

### SPM analysis of the pseudo-control group (group I)

Among the 24 children retained within this group, SPM-P analysis was completely negative in 15. Artifacts were found in the nine remaining children, but they were small (less than 200 voxels) and disseminated on the whole cortex (Table 2). By contrast, in the three additional children transferred from group I to group II because their PET images were found abnormal at SPM-step analysis and the artifacts included more than 400 voxels (1 child with one hypo-, 1 other child with one hyper-, and 1 with several hypo- and hypermetabolic clusters respectively).

**Table 2:** **Artifact localization in nine patients from group I (pediatric pseudo-control group, *n *= 24) using SPM-P procedure**

		**Hypometabolism**		**Hypermetabolism**	
Patients^a^	-	5/9	-	6/9	-
Location	Side	Clusters (voxels)^a^	*Z* max	Clusters (voxels)	*Z* max
Occipital	R	2 (76 to 188)	4.84	2 (68 to 78)	4.14
	L	1 (115)	4.49	8 (45 to 156)	4.22
Ins-temp	R	3 (59 to 181)	4.13	-	-
	L	2 (56 to 65)	3.56	3 (87–183)	4.19
Parietal	R	1 (66)	-	-	-
	L	-	-	-	-
Central	R	1 (68)	3.96	-	-
	L	-	1 (46)	3.67	-
Frontal	R	-	-	2 (126 to 175)	4.28
	L	-	-	1 (191)	4.60
Total	-	11	4.84	17	4.60

^a^numbers; R right; L left; Ins-temp insulo-temporal.

### SPM analyses of the patient group (group II)

Among the three *p* value thresholds tested, the best compromise between sensitivity and specificity was obtained for *p* < 0.001 for both SPM-A and SPM-P analyses (Table 3). Among the 3 voxel extents tested, the best compromise between sensitivity and specificity at *p* < 0.001 was obtained for 40 voxels for both SPM-A and SPM-P analyses (Table 3). These two thresholds were therefore selected for the following results.

**Table 3:** Sensitivity and (specificity) of SPM-A and SPM-P analysis using the different thresholds

**Voxel extent**	**>40 voxels**			**>100 voxels**			**>200 voxels**		
SPM_*p*_	*p* < 0.0001^a^	*p* < 0.001^a^	*p* < 0.05^b^	*p* < 0.0001^a^	*p* < 0.001^a^	*p* < 0.05^b^	*p* < 0.0001^a^	*p* < 0.001^a^	*p* < 0.05^b^
SPM-A	0.60 (0.97)	0.87 (0.89)	0.62 (0.92)	0.41 (0.99)	0.82 (0.86)	0.56 (0.93)	0.39 (0.99)	0.71 (0.94)	0.56 (0.75)
SPM-P	0.56 (0.98)	0.79 (0.97)	0.56 (0.97)	0.47 (0.99)	0.73 (0.97)	0.55 (0.98)	0.39 (0.99)	0.62 (0.98)	0.59 (0.99)

^a^Uncorrected; ^b^corrected.

SPM-A analysis found significant hypometabolic clusters in 120 (25%) out of the 470 cortical regions studied: 78/120 (65%) corresponded to true hypometabolic clusters (74 were concordant with visual analysis; for the remaining four, the hypometabolic cluster was clinically relevant although visually normal) and the remaining 42 were hypometabolic artifacts. By contrast, SPM-A failed to detect any hypometabolic cluster in 12 regions which were hypometabolic on visual analysis. As a result, our SPM-A procedure had a sensitivity of 87%, specificity of 89%, positive predictive value of 65%, and negative predictive value of 95%.

SPM-P analysis found significant hypometabolic clusters in 83 (18%) out of the 470 cortical regions studied: 71/83 (86%) corresponded to true hypometabolic clusters (68 were concordant to visual analysis, the remaining three were visually normal but clinically relevant), and the remaining 12 were hypometabolic artifacts. By contrast, SPM-P failed to detect any hypometabolic cluster in 19 regions which were hypometabolic on visual analysis. As a result, our SPM-P procedure had a sensitivity of 79%, specificity of 97%, positive predictive value of 86%, and a negative predictive value of 95%.

**Figure 3:**
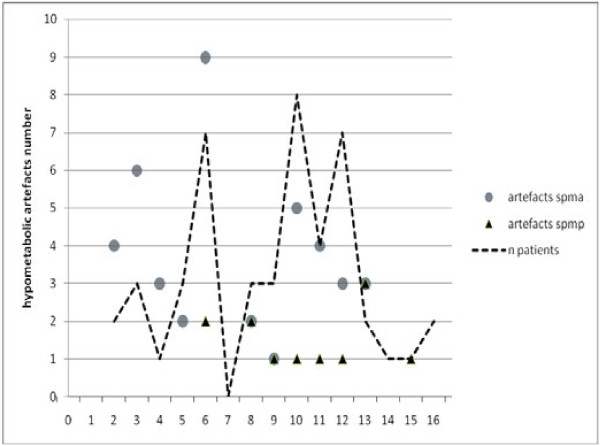
**Hypometabolic artifacts observed in patient group using SPM-A and SPM-P analysis.** Hypometabolic artifacts observed in patient group (*n* = 47) using SPM-A analysis (adult control group) and SPM-P analysis (pediatric pseudo-control group). *X* axis presents the ages (in years). *Y* axis presents the number of hypometabolic artifacts (false positive results) using SPM-A (lozenges) and SPM-P (circles) analysis. The broken line represents the number of patients at the different ages. Notice that artifacts are observed less frequently using SPM-P than SPM-A, and none was found in patients under 6 years old using SPM-P.

Sensitivity was moderately better using SPM-A than SPM-P (*p* = 0.039, Mac Nemar test). By contrast, specificity was dramatically higher using SPM-P than SPM-A (*p* < 0.0001). This was due to the higher positive predictive value using SPM-P, which markedly reduced the number of hypometabolic artifact detected, whereas the negative predictive value was equivalent using both procedures. Comparing SPM-A and SPM-P procedures, the number of artifacts tended to be less in younger children (especially under 6 years of age) using SPM-P (Figure 3). Additional artifacts were found within the interhemispheric fissure: 13 when using SPM-A, of which only 4 persisting when using SPM-P.

Artifacts did not result exclusively from the normalization procedure; the age-related changes may also play a role. Indeed, artifacts did not disappear when analyzing pediatric patients using the adult control group normalized with the pediatric template; their number decreased when compared to SPM-A procedure, but increased compared to SPM-P.

### Concordance of analysis procedures

**Figure 4:**
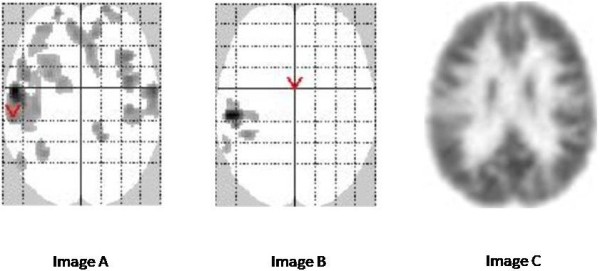
**Comparative results using SPM-A, SPM-P, and visual analyses.** Comparative results using SPM-A (adult control group), SPM-P (pediatric pseudo-control group) and visual analyses in a 4 year old patient. In this 4-year-old patient with positive MRI and seizure onset zone (SOZ) in the left *insula*, visual analysis (image **C**) shows a left insular hypometabolic area (axial slice). SPM-P (image **B**, SPM glass brain) identifies a hypometabolic cluster in the same area. SPM-A (image **A**, SPM glass brain) also identifies this cluster, but also shows several additional hypometabolic artifacts mainly located within the frontal cortex, ipsi- and contralaterally.

Visual analysis, SPM-P and SPM-A were concordant in 36 patients (77%), being all negative in 3 of them, and positive in the remaining 33 (80% of the 41 patients with positive visual analysis) (Table 4). Concordance was complete in 28/33 (68%), with the most significant true hypometabolic cluster on SPM having the same lobar and intra-lobar location and including the same number of regions whatever the method of analysis (Figure 4). Concordance was partial in the remaining five patients, with more hypometabolic regions detected with visual analysis in four and with SPM in one. Visual analysis, SPM-P, and SPM-A were discordant in the remaining 11 patients (23%). In two cases both SPM procedures disclosed the same true hypometabolic cluster, but visual analysis was negative. In nine cases SPM-P did not detect any abnormality whereas visual analysis, SPM-A, or both did in respectively 3, 1, and 5 cases with the same localization. Finally, kappa test gave a significant concordance of 0.448 ± 0.084 (*p* < 0.005) between the three analysis procedures, 0.598 ± 0.140 (*p* < 0.005) between both SPM methods, and 0.427 ± 0.194 (*p* < 0.005) or 0.330 ± 0.158 (*p* = 0.007) between visual analysis and respectively, SPM-A or SPM-P.

**Table 4:** Patients with at least one true hypometabolism using visual, SPM-P, and SPM-A analysis

		**Visual analysis**	
**SPM-P**	**SPM-A**	**Positive**	**Negative**
Positive	Positive	33 (17C, 16S)	2 (2C, 0S)
Negative	Positive	5 (4C, 1S)	1 (0C, 1S)
	Negative	3 (3C, 0S)	3 (1C, 2S)
Total		41 (24C, 17S)	6 (3C, 3S)

SPM-P, SPM analysis using the pediatric pseudo-control group; SPM-A, SPM analysis using the adult control group; C, cryptogenic (MRI-negative) patients; S, symptomatic (MRI-positive) patients.

Regarding the 20 MRI-positive patients, the three procedures were concordant in 18 (90%) (kappa = 0.739 ± 0.129; *p* < 0.005), being all positive in 16 and all negative in two, and they were discordant in the remaining two with negative SPM-P. Concordance between visual analysis and SPM procedure reached 0.773 ± 0.216 (*p* < 0.005) for SPM-A and 0.828 ± 0.166 (*p* < 0.005) for SPM-P. Regarding the 27 MRI-negative patients, the three procedures were concordant in 18 (67%) (kappa = 0.264 ± 0.111; *p* < 0.01), being all positive in 17 and all negative in one, and they were discordant in the remaining nine with negative SPM-P in seven of them. Concordance between visual analysis and SPM procedures did not reach significance.

## Discussion

The goal of this study was to evaluate the performances of SPM analysis of FDG-PET in children using an age-matched pseudo-control group of epileptic patients with robustly negative imaging versus classical healthy young adult controls. Both SPM analyses achieve a similar rate of detection of hypometabolic clusters and perform as well as visual analysis, not only in MRI-positive patients but also in MRI-negative ones. Although the sensitivity of SPM is not increased using pediatric vs. adult controls (79% vs. 87%), specificity is increased (97% vs. 89%) due to the reduction of the number of hypometabolic artifacts detected. As a result the positive predictive value of the method rises from 65% to 86%. That provides a relevant clinical impact especially in the younger children.

Visual inspection of FDG-PET images is extensively used in clinical epilepsy practice and remains the reference method of analysis in children [14,27]. The hypometabolic areas detected have been proved to include the seizure onset zone, although they usually are more extended and do not delineate it [6,8]. The localizing value of visual PET analysis varies from 36% to 73% in extratemporal cases, depending whether MRI is negative or not [9,20], and reaches 90% exclusively in temporal lobe cases [26]. This detection value can be improved, particularly in extratemporal epilepsy with negative MRI, using PET/MRI coregistration [12,13]. That has been confirmed by intracranial EEG and post-operative data. The present 56% detection rate of relevant hypometabolic areas is therefore in accordance with the literature, if one considers that we did not use coregistration and that the series mostly comprises MRI-negative patients and children with extratemporal epilepsy. In addition, the ability to detect a hypometabolic area visually is subjective and depends on the expertise and experience of the observer. Most reported series tend to limit this bias by achieving a consensus between closely performing readers, as we presently did with a first concordance of 0.81.

By contrast, SPM analysis is observer independent [1,2]. Taking the whole brain into account, it allows assessing the spatial extent and location of the abnormal site on the brain map with no a priori hypothesis. Initially dedicated to the comparison of data sets among groups of subjects, SPM method also proved to be reliable for quantitative analysis of individual FDG-PET scans in various neurological disorders, including epilepsy [37]. SPM analysis has been widely applied to the study of adults with epilepsy, with thresholds varying from *p* < 0.001 (uncorrected) to *p* < 0.05 (corrected) and >20 voxels to >250 voxels [5,7]. Since SPM showed comparable sensitivity to visual assessment, it is considered as an aid in diagnosing seizure onset zones in temporal as well as in extratemporal lobe epilepsy and in lesional as well as in non-lesional cases [5,7,12,15,16,38].

In children, experience with SPM in epilepsy is more limited. Only one study formally assessed the role of statistical threshold on sensitivity and specificity [27]: they found *p* < 0.001 as the best compromise, as we presently do. Based on young adults as controls, the results are not univocal. De Tiege et al. did succeed detecting areas of remote inhibition in children aged 5 to 11 years with a particular epileptic encephalopathy and to see them vanish when epilepsy and cognitive deficits recover [24,39,40]. Some authors showed a high SPM performance (86%) for correct localization of the seizure onset zone in adolescents (12 to 15 years) [26], but others achieved a rate of only 13% in children from the age of 3 [14]. In another series, the same procedure gave an SPM sensitivity of 74% in children over 6 years old [27], whereas it failed in children under 6 years of age due to a significant proportion of artifacts [23]. We also found a high number of such false positive hypometabolic clusters when using an adult template, at a threshold stringent for such an individual SPM analysis [12]. One main issue is the different sizes of the head of adults and young children [23]. We confirm here that this artifact rate is higher in the younger ages and can be reduced by using a pediatric template [36]. Another potential advantage of a pseudo-normal epilepsy group could be that the potential effect of the antiepileptic drugs is largely cancelled out since both the patients and the control groups were on antiepileptic medication [35]. To generate even better templates, one could speculate on data from other pathologies that justify whole-body PET scanning but may respect the brain, like pediatric lymphoma, for example [41].

In order to deal with the pediatric controls the closest to “normals”, we confirmed PET negativity using the Signorini’s method [37]: each data set of the pseudo-control group was compared to the rest of the group using SPM. The complete procedure excluded three additional children. Our main result is a gain of specificity and this *pediatric* procedure reaches the highest rates ever reported in epilepsy for SPM. Conversely, the predictive positive value is improved, thus decreasing the number of artifacts and optimizing the clinical relevance of SPM analysis compared to the classical *adult* procedure. We assume that these advantages of pediatric SPM can compensate the slightly decreased sensitivity compared to adult SPM (79% vs. 87%). Note that these rates of sensitivity and specificity apply to SPM analysis and not to FDG-PET in general, since we excluded from their calculation the major part of negative cases. Nevertheless, this method was robustly able to identify some clinically relevant hypometabolic areas missed by visual analysis in 6% of cases.

Although our template presents with the usual balance of specificity versus sensitivity, we recently showed the whole procedure to be beneficial also in multifocal childhood epilepsy when visual PET analysis fails to detect bilateral abnormalities, as in school-age children with a fever-induced epileptic encephalopathy [42].

## Conclusion

Such a pediatric SPM procedure is a way to optimize SPM analysis of FDG-PET in children with epilepsy, useful for clinical purpose and for research. It might also be considered for other brain pathologies in pediatrics in the future.

### Statement

Authors propose to make their pseudo-control pediatric group available to the neuroscientific community, free, according to a statement of agreement with the CEA regarding the context of use for their data basis.

## Competing interests

The authors declare that they have no competing interest.

## Authors’ contributions

FA performed most of the PET examinations, the visual analysis, the SPM analysis, coordinated the results and contributed to draft the manuscript. VB performed some PET examinations, participated in its design and helped to draft the manuscript. LHP read MRI examinations. PCR performed the statistical analyses. SR participated in the design of the study. OD followed and referred the patients and extensively reviewed the manuscript. FC performed PET visual analysis and reviewed the manuscript. CC conceived the study design and drafted the manuscript. All authors read and approved the final manuscript.
